# Growth Hormone Stimulation Testing: To Test or Not to Test? That Is One of the Questions

**DOI:** 10.3389/fendo.2022.902364

**Published:** 2022-06-09

**Authors:** Mabel Yau, Robert Rapaport

**Affiliations:** Division of Pediatric Endocrine and Diabetes, Mount Sinai Kravis Children’s Hospital, New York, NY, United States

**Keywords:** sex hormone priming, IGF – I, short stature, growth hormone deficiency, growth hormone – secretion

## Abstract

The evaluation of children with short stature includes monitoring over a prolonged period to establish a growth pattern as well as the exclusion of chronic medical conditions that affect growth. After a period of monitoring, evaluation, and screening, growth hormone stimulation testing is considered when the diagnosis of growth hormone deficiency (GHD) is entertained. Though flawed, growth hormone stimulation tests remain part of the comprehensive evaluation of growth and are essential for the diagnosis of growth hormone (GH) deficiency. Variables including testing length, growth hormone assay and diagnostic cut off affect results. Beyond the intrinsic issues of testing, results of GH stimulation testing can be influenced by patient characteristics. Various factors including age, gender, puberty, nutritional status and body weight modulate the secretion of GH.

## Introduction

Concern about poor growth is the leading reason for referrals to a pediatric endocrinologist (1). The evaluation of children with short stature includes monitoring over a prolonged period to establish a growth pattern as well as the exclusion of chronic medical conditions that affect growth. After a period of monitoring, evaluation, and screening, growth hormone stimulation testing is considered when the diagnosis of growth hormone deficiency (GHD) is entertained.

Lack of puberty at an expected age is another common reason patients are referred. Typically, 95% of girls present at least one sign of puberty by 13 years of age and boys by 14 years of age. These conditions of growth and pubertal delay can be difficult to diagnose because the rate of growth appears to decelerate as they cross growth percentiles around the time of the anticipated pubertal growth spurt. Thus, the growth deceleration due to constitutional delay of puberty and growth hormone deficiency can be difficult to ascertain with certainty (1).

Measuring GH concentrations at random times is unreliable because growth hormone (GH) is secreted from the anterior pituitary in a pulsatile fashion and is mainly stimulated by the release of hypothalamic growth hormone-releasing hormone (GHRH). Instead, patients are usually screened for GH deficiency by measuring serum IGF-1 and IGF-BP3 levels which have longer half-life and no pulsatility. Though flawed, growth hormone stimulation tests remain part of the comprehensive evaluation of growth and are essential for the diagnosis of growth hormone (GH) deficiency (2–4). For GH stimulation testing, two agents provoke GH secretion from the pituitary (L-dopa, clonidine, arginine, glucagon). These provocative agents are not physiological and do not replicate normal secretory dynamics. The insulin tolerance test which is considered the gold standard for diagnosis of GHD is used to assess GH secretion in response to hypoglycemia. Given the risks associated with hypoglycemia, it is performed less frequently in the outpatient setting. Serial blood samples are taken to detect the point of maximal serum concentration of GH (1). Peak growth hormone (PGH) response to provocative testing is a vital determinant of the clinical response to GH therapy. In a study by Cohen et al. of prepubertal children characterized as GH deficient or idiopathic short stature based on GH stimulation testing without sex hormone priming, those with idiopathic short stature required doses nearly 2 times higher to reach an IGF-1 target of 2 SDS (5). The stimulated PGH level response to two pharmacologic stimuli that distinguishes between GH deficient and sufficient patients is unclear and likely exists on a continuum with levels of 5, 7 and 10 ng/mL having been proposed, each without adequate data for substantiation. The currently agreed upon peak GH cutoff is 10 ng/mL (1, 6, 7). Stimulation testing with arginine and levodopa with samples obtained for 3 hours is perhaps most frequently used in many large multicenter studies (3, 6, 8, 9).

## Intrinsic Test Factors

### Test Length

Some studies of various GH ST protocols suggest that sampling or duration can be reduced from 3 hours, whilst preserving the accuracy of diagnosis (10–13). Data from our single center experience of provocative GH testing using an identical protocol on a large cohort of 315 pediatric patients with short stature and/or growth failure showed peak GH response was reached by 2 hours in 97.8% of those tested. This study indicated that the GH ST with arginine and levodopa can be terminated at 2 hours without compromising its diagnostic value based on the currently accepted peak growth hormone response cutoff of 10 ng/mL, as exclusion of the 3 hour sample did not alter the GH sufficiency status in any of the 315 patients (9).

### Growth Hormone Assay

There are several practical and logistical limitations to stimulation testing. Results are often often vary depending on which assay is used to analyze blood samples. Historically, GH was measured by a wide variety of approaches including bioassays, radio receptor assays, immunoassays and mass spectrometry (14). Currently, immunoassays are used most commonly to measure serum GH concentrations in clinical settings. Endogenous GH in serum exists in numerous isoforms with the majority being the isoform of 22 kDa molecular weight. However, approximately 10% circulates as the 20 kDa isoform and other isoforms and growth hormone fragments circulate in smaller portions (15). Different immunoassays can detect different spectrums of total GH isoforms. In an effort to standardize across isoforms, current consensus guidelines recommend assay calibration with a highly purified preparation of the 22 kDa recombinant human GH isoform of GH (2, 16). Additionally, GH immunoassays transitioned from using polyclonal antibodies that targeted multiple epitopes on varying GH isoforms to monoclonal antibodies targeting one isoform (14). With these changes, current assays have a narrower target.

On the new assays, GH concentrations yields are lower than on older assays. Cutoffs for peak growth hormone response to GH stimulation testing may need to be revisited with the adoption of newer assays with lower reported GH concentrations. Since only small changes in isoform ratios have been reported in certain states such has pituitary tumors and exercise, concentrations of 22 kDa GH accurately reflect total GH secretion (14). Still, inter-assay differences between immunoassays occur due to differences variations in the type of immunoassay, antibody specificity, and interference from GH binding proteins (14).

### Peak Growth Hormone Cut Off

As recombinant growth hormone became more widely available, less stringent criteria for the diagnosis of GHD were implemented with increase in peak GH cut off levels. With the renewed interest in oral GH secretagogues, a reassessment of peak GH cut offs may be helpful. The studies by Bright et al. and Blum et al. suggest that a partially intact pituitary axis is needed for GH secretagogues to be effective (17, 18). Individuals with “moderate” growth hormone deficiency who may respond to GH secretagogues need to be differentiated from those with “severe” growth hormone deficiency who require growth hormone therapy (9).

Traditionally, the interpretation of GH stimulation testing results was binary with the adherence to pass/fail diagnostic GH cutoffs. Perhaps, instead, the results should be interpreted on a continuum that spans severe GHD requiring GH therapy to moderate or provisional GHD for which alternative therapies and further monitoring of growth should be considered (19). There is increasing evidence supporting the need to revisit cutoffs for peak GH after stimulation based on the assay used to measure serum GH concentrations (20, 21). Lower cutoffs for peak GH levels based on specific assays have been proposed. The establishment of method-specific clinical evidence-based GH cutoff limits would help ensure adequate clinical diagnoses.

### Supporting MRI Findings

In GHD, brain MRI may show congenital pituitary abnormalities such as anterior pituitary dysplasia/hypoplasia, pituitary stalk interruption syndrome, and developmental cyst but also tumoral lesions (22). Neuroimaging is a crucial study in the diagnostic process of GHD. With only partial integrity of the hypothalamic pituitary connections, growth hormone secretion was able to be stimulated by growth hormone releasing hormone plus Arginine (23). In children with congenital GHD but less severe impairment of the pituitary stalk, the GH response to stimulation may be sufficient but pituitary GH reserve deteriorates with a GH response of < 10 ug/L after 20 yr of age (23). MRI may be helpful in differentiating those with moderate or provisional GHD. Findings of pituitary abnormalities support decisions on GH treatment in such cases of moderate GHD (peak GH of 7-10 ng/ml), as GHD is expected to evolve.

### Patient Factors

Beyond the intrinsic issues of testing, results of GH stimulation testing can be influenced by patient characteristics. GH secretion is influenced by several factors including age, gender, puberty, nutritional status and body weight (24–26).

### BMI

Obesity has been associated with decreased spontaneous and stimulated GH secretion in both adults (27–30), and children (31) and weight reduction has been followed by increased GH secretion (31–35). Though the exact neuroendocrine mechanism causing the blunted GH response in obesity is unknown, proposed mechanisms include high circulating levels of insulin which can suppress GH synthesis and release and adipocyte-secreted leptin affecting GH regulation (36–39). It has been demonstrated that PGH response to stimulation testing with A-LD decreased with higher BMI SDS in a large cohort of normal weight healthy children with a range of BMI that approximated a normal distribution (mean BMI SDS of -0.3 ± 1.0). This finding suggests that the inverse relationship between BMI and PGH is not isolated to obesity and is evident in the normal weight children (9). Still, BMI is not currently consistently considered in the interpretation of the peak GH response in children.

### Puberty and Sex Hormones

During puberty, there is a normal increase in growth hormone concentrations due to a larger mass of GH released per pituitary secretory episode resulting from a higher maximal rate of GH secretion per secretory burst (40). Due to the physiologic rise of GH during puberty, there is a debate as to whether prepubertal children should be “primed” with sex hormones before GH provocative testing (1). In addition to endogenous sex hormones, short term administration of exogenous sex hormones can modulate growth hormone secretion (41). Priming leads to increased peak GH levels and decreases the false positive rate for diagnosing GH deficiency in healthy controls (42–44). In the study by Marin et. al, a subset of 11 prepubertal normal children were primed with 2 days of estrogen. Peak GH response rose to levels seen in subjects at pubertal stages 4 and 5 (45). In a later study by Muller et al. of 26 boys primed with a single dose of testosterone, 77% increased their peak GH level to > 10 ng/ml (46). In a study of 315 patients undergoing GH stimulation testing, there was no difference in rates of GHD in prepubertal and pubertal patients (9).

In 2016, the Pediatric Endocrine Society updated their guidelines to support priming with sex hormones in prepubertal children (boys > 11 years old and girls > 10 years old) (2). The stated reason for this recommendation of priming was to avoid unnecessary GH treatment of children with constitutional delay of growth and puberty (2). Yet, the practice of priming remains controversial in Europe (47). In a study of 8 European countries and the US performed after these guidelines were published, priming was recommended in 5 out of 9 countries (48).

This hormonal milieu of puberty is not sustained after priming. On these supraphysiologic testosterone levels, endogenous growth hormone secretion may be overestimated. Will these children who responded to exogenous sex hormones be able to secrete enough GH at the time of puberty? Would peripubertal children who have lower peak GH levels without priming benefit from exogenous GH therapy? This overestimation can lead to false negative results and deny eligible children required treatment with growth hormone. It is unclear whether children diagnosed with GHD with or without priming respond differently to GH treatment. However, constitutional delay and GHD can be difficult to differentiate and priming should be considered in delayed puberty (49). Short term adverse side effects of priapism and testicular pain were reported in approximately 3% of prepubertal boys primed with short courses of testosterone (50).

Altering a patient’s baseline characteristics is not recommended with any other stimulation testing to diagnose a hormonal deficiency. As an alternative to priming, normative values of peak growth hormone response should be further explored to develop cut off limits based on pubertal stage. This was first proposed by Rose et al. when they found that mean spontaneous night time growth hormone levels rose during pubertal development in both boys and girls, with the highest levels at mid-puberty (51). Currently, distinct cut offs are only defined for children and adults. Cut offs based on pubertal staging would bridge the continuum. Reassessment of the GH/IGF-I axis when a child treated with growth hormone peripubertally enters puberty has been proposed as another alternative to priming (19). Though it is common in practice to continue growth hormone therapy once diagnosed with GHD until the epiphyses close, GH therapy could be paused at onset of puberty to repeat the GH stimulation test and determine if continued therapy is necessary. We recommend obtaining pubertal hormone levels at time of GH stimulation testing to correlate GH response to pubertal status. In our clinical experience, we have cared for patients with low peak GH response to stimulation without priming in whom we elected not to treat that later demonstrated adequate growth velocity and adult height. This demonstrates the importance of the clinician’s interpretation of clinical findings in combination with stimulation testing results.

## Discussion

Given its flaws, one should enter GH ST with a high predictive value. The Pediatric Endocrine Society recommends against the use of GH stimulation testing as the sole diagnostic criterion of GHD (2). The decision to proceed with growth hormone (GH ST) stimulation testing should be reached only after careful consideration and only when the result will significantly contribute to the diagnostic process (9). If one combines stimulation testing result with the patient’s anthropometric measurements, height velocity, physical findings, screening tests, and IGF-1 and IGF-BP3 levels, more complete clinical picture is capture that allows for proper individualized diagnosis and treatment (1). While by itself growth hormone stimulation testing is unreliable, within the overall picture of a patient with short stature, decreased growth velocity, and low IGF-1 level, the results of growth hormone stimulation testing may complete a picture that the astute clinician can properly utilize to decide on interventions such as growth hormone therapy.

In conclusion we recommend careful, long term observation of patients with growth failure. The decision to undertake growth hormone stimulation should be reserved for those in whom its results would be the last and deciding parameter for therapeutic intervention. If still unclear, additional observation and evaluations such as genetic testing and perhaps repeat stimulation testing should be considered.

## Author Contributions

MY contributed with substantial contributions to the conception or design of the work; or the acquisition, analysis or interpretation of data for the work, and drafting the work or revising it critically for important intellectual content. RR contributed by providing approval for publication of the content and analysis or interpretation of data for the work, and drafting the work or revising it critically for important intellectual content. All authors contributed to the article and approved the submitted version.

## Conflict of Interest

The authors declare that the research was conducted in the absence of any commercial or financial relationships that could be construed as a potential conflict of interest.

## Publisher’s Note

All claims expressed in this article are solely those of the authors and do not necessarily represent those of their affiliated organizations, or those of the publisher, the editors and the reviewers. Any product that may be evaluated in this article, or claim that may be made by its manufacturer, is not guaranteed or endorsed by the publisher.
